# Alternative donor peripheral blood stem cell transplantation for the treatment of high-risk refractory and/or relapsed childhood acute leukemia: a randomized trial

**DOI:** 10.1186/s40164-020-00162-6

**Published:** 2020-04-06

**Authors:** Binglei Zhang, Jian Zhou, Fengkuan Yu, Tianxin Lv, Baijun Fang, Dandan Fan, Zhenyu Ji, Yongping Song

**Affiliations:** 1 School of Basic Medical Sciences, Zhengzhou University, Zhengzhou, 450000 Henan China; 2 Academy of Medical Sciences, Zhengzhou University, Zhengzhou, 450000 Henan China; 3 Department of Hematology, Affiliated Cancer Hospital of Zhengzhou University and Henan Cancer Hospital, Zhengzhou, 450000 Henan China; 4 Henan Academy of Medical and Pharmaceutical Sciences, Zhengzhou University, Zhengzhou, 450000 Henan China

**Keywords:** Prognosis, High-risk refractory and/or relapsed childhood acute leukemia, Allogeneic stem cell transplantation, Matched sibling donors, Alternative donors

## Abstract

**Background:**

The high-risk refractory and/or relapsed (R/R) childhood acute leukemia prognosis is poor, and allogeneic stem cell transplantation (allo-HSCT) is the most prudent treatment modality. However, there are limited matched sibling donors (MSDs), and alternative donors (ADs) are the main source for allo-HSCT. Thus, we evaluated the clinical efficacy of AD peripheral allo-HSCT for treating high-risk R/R childhood acute leukemia.

**Methods:**

We assessed 111 children who underwent allo-HSCT at the Affiliated Cancer Hospital of Zhengzhou University between October 2006 and July 2019. The patients were divided in the MSD and AD groups, and their clinical characteristics, complications, and survival rates were compared.

**Results:**

The cumulative incidences of Epstein–Barr virus and cytomegalovirus infections were significantly higher in the AD than in the MSD group (*P *< 0.001); however, the recurrence and mortality rates were significantly higher in the MSD than in the AD group (*P *< 0.05). Furthermore, the 5-year disease-free (DFS) (65.2% vs. 43.3%, *P *= 0.033) and overall survival (OS) rates (71.6% vs. 53.8%, *P *= 0.053) were significantly higher in the AD than in the MSD group. In the AD group, the grade II–IV acute graft-versus-host disease (aGVHD), donor-recipient ABO compatibility, conditioning regimen, and CMV infection affected the 5-year OS. The grade II–IV aGVHD also affected the 5-year DFS; however, only the donor-recipient ABO compatibility affected the 5-year DFS. The donor MSD (HR: 2.035, 95% confidence interval [CI] 1.057–3.920, *P *= 0.034) and the grade II–IV aGVHD (HR: 2.914, 95% CI 1.261–6.736, *P *= 0.012) affected the 5-year DFS of childhood acute leukemia after allo-HSCT, and the grade II–IV aGVHD (HR: 3.016, 95% CI 1.217–7.473, *P *= 0.017) affected the 5-year OS. Moreover, the donor source (HR: 2.836, 95% CI 1.179–6.823, *P *= 0.020) and grade II–IV aGVHD (HR: 3.731, 95% CI 1.332–10.454, *P *= 0.012) were independent predictors of the 5-year DFS, while the latter (HR: 3.524, 95% CI 1.310–10.988, *P *= 0.030) was an independent predictor of the 5-year OS.

**Conclusions:**

AD-PBSCT was effective for high-risk R/R childhood leukemia and may have better clinical outcomes than MSD-PBSCT; thus, it can be used as first-line treatment for high-risk R/R childhood leukemia.

## Background

Acute leukemia has become a common malignancy among children, and the incidence has increased gradually. Particularly, acute lymphoblastic leukemia (ALL) has been the most common tumor among children worldwide and in the Middle East [1]. Approximately 98% of children with ALL can attain remission, and approximately 85% of patients younger than 18 years with newly diagnosed ALL treated with chemotherapy have a 5-year survival probability of 90% [2, 3]. Although approximately 20% of patients experience disease relapse [4], the survival rate is still quite poor after relapse, especially among patients with high risk [5], and the long-term survival rate is less than 60% [6]. Acute myeloid leukemia (AML) is a type of malignant hematological disease with strong heterogeneity and a great variety of effects. Despite great progress in its diagnosis and treatment, the mortality rate of AML remains relatively high and threatens patients’ lives severely, and the 5-year overall survival (OS) rate among patients younger than 19 years is approximately 65% [7, 8]. In addition, high-risk patients with ALL and AML usually have poorer prognosis with routine treatment. Thus, allogeneic hematopoietic stem cell transplantation (allo-HSCT) is ultimately needed for high-risk patients with refractory and/or relapsed (R/R) disease and may be the only cure.

Allo-HSCT has been widely used as the treatment for hematological diseases. The efficacy of matching-sibling bone marrow transplantation is very significant; however, less than 30% of patients have matching-sibling donors (MSDs) [9–11] and the collection of bone marrow is relatively cumbersome. Due to the establishment and development of bone marrow donors worldwide, the source of alternative donors (ADs) is relatively broad. With the continuous improvement of conditioning regimen and supportive therapy, the efficacy of AD peripheral blood stem cell transplantation (AD-PBSCT) has also been continuously improved. Therefore, PBSC may be the main source of stem cells for allo-HSCT in future.

AD-PBSCT as the primary treatment for childhood acute leukemia remains controversial. Our study compared AD-PBSCT with MSD-PBSCT among patients with high-risk R/R childhood leukemia regarding the effects, complications, and influencing factors, providing a theoretical basis for the therapeutic role of AD-PBSCT in this patient population.

## Methods

The study protocol was approved by the ethics committee of our hospital, it was performed in accordance with the Declaration of Helsinki, and the guardians of all the patients provided informed consent for their inclusion at the Affiliated Cancer Hospital of Zhengzhou University. The basic medical record data of 111 patients were retrieved and analyzed (Table 1). All patients were diagnosed and reevaluated according to the National Comprehensive Cancer Network Clinical Practice Guidelines (2012 version) [12, 13]. The definition of high-risk/refractory pediatric acute leukemia is based on the European Society for Blood and Marrow standards [14, 15]. The patients underwent allo-HSCT between October 2006 and July 2019. There were no differences in collection procedure and age between the ADs and MSDs. The inclusion criteria were: (1) acute leukemia (AML or ALL), (2) age ≤ 14 years at the time of allo-HSCT, (3) high-risk R/R diagnosis, and (4) PBSCT performance.

**Table 1:** Characteristics of all patients between MSD and AD

Variables	MSD(n = 42)	AD(n = 69)	χ^2^	*P* value
Gender (n, %)			0.172	0.678
Male	27 (64.3)	47 (68.1)		
Female	15 (35.7)	22 (31.9)		
Primary disease (n, %)			0.016	0.899
AML	22 (52.4)	37 (53.6)		
ALL	20 (47.6)	32 (46.4)		
Disease status at HSCT (n, %)			1.873	0.392
First CR	19 (45.2)	39 (56.5)		
Second and other CR	16 (38.1)	18 (26.1)		
Relapse	7 (16.7)	12 (17.4)		
Disease status at HSCT (n, %)			1.332	0.248
First CR	19 (45.2)	39 (56.5)		
Other	23 (54.8)	30 (43.5)		
Extramedullary infiltration (n, %)			2.538	0.111
Yes	8 (19.0)	6 (8.7)		
No	34 (81.0)	63 (91.3)		
Conditioning regimen (n, %)			0.082	0.775
Bu/Cy-based	30 (71.4)	51 (73.9)		
TBI/Cy-based	12 (28.6)	18 (26.1)		
Gender of donor-recipient (n, %)			5.669	0.017
Identical	19 (45.2)	47 (68.1)		
Different	23 (54.8)	22 (31.9)		
Donor-recipient ABO compatibility (n, %)			0.014	0.907
Compatible	19 (45.2)	32 (46.4)		
Incompatible	23 (54.8)	37 (53.6)		
Abnormal markers			NA	NA
t(9;22)	8 (19.0)	13 (18.8)		
MLL/AF4	1 (2.4)	3 (4.3)		
FLT3/ITD	2 (4.8)	1 (1.4)		
MRD			0.014	0.906
Yes	12 (28.6)	19 (27.5)		
No	30 (71.4)	50 (72.5)		
MNC (× 10^8^/kg)	11.74 (3.98–31.03)	14.57 (3.57–47.11)	NA	NA
CD34 + cells(× 10^6^/kg)	5.84 (3.43–23.30)	8.33 (3.07–35.7)	NA	NA
Time for implantation of neutrophils (d)	12 (9–19)	13 (10–26)	NA	NA
Time for implantation of PLT(d)	13 (8–30)	13 (9–26)	NA	NA

*MSD* matched sibling donor, *AD* alternative donor, *AML* acute myeloid leukemia, *ALL* acute lymphoblastic leukemia, *HSCT* hematopoietic stem cell transplantation, *CR* complete remission, *BU* busulfan, *TBI* total body irradiation, *Cy* cyclophosphamide, *MRD* minimal residual disease *MNC* mononuclear cells, *PLT* platelet, *NA* not applicable

The entire study cohort was divided into the MSD and AD groups based on the donor source. The MSD group (42 patients) matched completely at the human leukocyte antigen (HLA) 10/10 or HLA 6/6 alleles (HLA-DR, HLA-DQ, HLA-A, HLA-B, and HLA-C by high-resolution type; HLA-A, HLA-B, and HLA-C by low-resolution type). The AD group (69 patients) included 32 unrelated and 37 relative haploidentical donors. Nineteen patients matched completely at the HLA10/10 alleles (HLA-DR, HLA-DQ, HLA-A, HLA-B, and HLA-C by high-resolution type) among the unrelated donors.

In the present study, the conditioning regimens among patients were busulfan (Bu) and cyclophosphamide (CTX)-based regimens (Bu/Cy-based, Bu 0.8 mg/kg q 6 h × 4 days, CTX 40–60 mg/kg × 2 days) and total body irradiation (TBI) combined with CTX-based regimens (TBI/Cy-based, TBI 4–5 Gy × 2 days, CTX 40–60 mg/kg × 2 days). To prevent graft-versus-host disease (GVHD), the MSD group was administered cyclosporine A (CsA) combined with a short course of low dose methotrexate and the AD group was administered mycophenolate mofetil and rabbit anti-human thymocyte immunoglobulin based on MSD. The plasma concentration of CsA was assessed every 3 days and maintained within 200–400 ng/mL. All patients were provided with timely and comprehensive support for symptomatic treatment, including the prevention of infection and hemorrhagic cystitis, the use of granulocyte colony-stimulating factor, and infusion of blood products.

The criterion for neutrophil implantation was a neutrophil count of ≥ 0.5 × 10^9^/Lon the first day and maintained for 3 consecutive days. The criterion for platelet implantation was a platelet count of ≥ 20 × 10^9^/L on the first day and maintained for 7 consecutive days without transfusion. After hematopoietic reconstitution, a bone marrow specimen was collected and assessed for evidence of implantation by quantitative polymerase chain reaction assay or sex chromosome analysis. Disease relapse was defined as hematological and clinical recurrence of leukemia. Death other than that due to disease relapse was considered non-relapse mortality. OS was considered the time from the receipt of allo-HSCT to death or the end of follow-up. Disease-free survival (DFS) was considered from the receipt of allo-HSCT to relapse, death, or end of follow-up. Follow-up was performed via outpatient (eight patients) or inpatient (98 patients) visits or via telephone (five patients). Some patients chose to be checked at the local hospital due to some special reasons, therefore, we informed the relevant inspection items in advance and acquired the results via telephone.

The classification data were represented as composition ratios. The count data were compared using the Chi squared or the Fisher’s exact test, as appropriate. The impacts of factors on survival time were compared using the log-rank test. Univariate analyses of OS and DFS were performed via the Kaplan–Meier method. The Cox regression model was used for multivariate survival analysis. All statistical analyses were performed using GraphPad Prism 7.0 (GraphPad Software, La Jolla, CA, USA) and SPSS 21.0 software (IBM Corp., Armonk, NY, USA). All statistical tests were two-tailed with statistical significance established at *P *< 0.05.

## Results

### Clinical characteristics

The clinical characteristics of patients are summarized in Table 1. The MSD group included 27 male and 15 female, 22 of whom were diagnosed with AML and 20 with ALL(of which 19 and 11 cases were refractory, respectively). The AD group included 47 male and 22 female, 37 of whom were diagnosed with AML and 32 with ALL(of which 24 and 12 cases were refractory, respectively). The number of patients who underwent allo-HSCT at the first complete remission (CR1), second, or other CR (all patients who achieved CR except CR1), and relapse were 19, 16, and 7, respectively, in the MSD group and 39, 18, and 12, respectively, in the AD group. Eight and six patients experienced extramedullary infiltration including relapse before transplantation in the MSD and AD groups, respectively. Seventy-nine patients were evaluated for minimal residual disease (MRD) by flow cytometry, and 31 patients had MRD pre-PBSCT (12 and 19 in the MSD and AD groups, respectively). The median numbers of transfused mononuclear cells were 11.74 (3.98–31.03) × 10^8^/kg and 14.57 (3.57–47.11) × 10^8^/kg, and the median numbers of transfused CD34^+^ cells were 5.84 (3.43–23.30) × 10^6^/kg and 8.33 (3.07–35.7) × 10^6^/kg in the MSD and AD groups, respectively. There were no significant differences in the basic clinical characteristics between the MSD and AD groups, apart from the gender among recipients.

### Engraftment and complications

Hematopoietic reconstruction was successfully performed in 109 patients, and two patients in the AD group experienced failure due to early graft rejection. The hematopoietic reconstitution rates among patients with AD and MSD were 98.2% and 100%, respectively. The median times for neutrophil implantation were at day 12 (range, days 9–19) and day 13 (range, days 10–26), respectively, while the corresponding for platelet implantation were days 13 (range, days 8–30) and 13 (range, days 9–26) in the MSD and AD groups, respectively. Acute GVHD (aGVHD) [16] and chronic GVHD (cGVHD) [17] were diagnosed and graded by referring to the Seattle standard and the consensus of the National Institutes of Health [18]. There were no significant differences in the cumulative incidences of aGVHD, cGVHD, invasive pulmonary fungal disease (IPFD), and hemorrhagic cystitis between the MSD and AD groups (all *P *> 0.05). The cumulative incidence of grade II–IV aGVHD was higher for the AD than for the MSD group, but without statistical significance (*P *= 0.052). The cumulative incidences of Epstein–Barr virus (EBV) and cytomegalovirus (CMV) infections were significantly higher in the AD than in the MSD group (all *P *< 0.001) (Table 2).

**Table 2:** Complications and prognosis of all patients between MSD and AD

Variables	MSD (n = 42)	AD (n = 69)	χ^2^	*P*-value
Acute GVHD (n, %)			0.435	0.509
Yes	15 (35.7)	29 (42.0)		
No	27 (64.3)	40 (58.0)		
Grade II-IV aGVHD (n, %)			3.788	0.052
Yes	2 (4.8)	12 (17.4)		
No	40 (95.2)	57 (82.6)		
Chronic GVHD (n, %)			0.046	0.83
Yes	9 (21.4)	16 (23.2)		
No	33 (78.6)	53 (76.8)		
IPFD (n, %)			0.274	0.601
Yes	12 (28.6)	23 (33.3)		
No	30 (71.4)	46 (66.7)		
Hemorrhagic cystitis (n, %)			3.690	0.055
Yes	8 (19.0)	25 (36.2)		
No	34 (81.0)	44 (63.8)		
EBV infection (n, %)			17.674	< 0.001
Yes	1 (2.4)	26 (37.7)		
No	41 (97.6)	43 (62.3)		
CMV infection (n, %)			13.100	< 0.001
Yes	9 (21.4)	39 (56.5)		
No	33 (78.6)	30 (43.5)		
Relapse (n, %)			5.056	0.025
Yes	19 (45.2)	17 (24.6)		
No	23 (54.8)	52 (75.4)		
Death (n, %)			4.197	0.041
Yes	16 (38.1)	14 (20.3)		
No	26 (61.9)	55 (79.7)		
Non-relapse mortality (n, %)			4.051	0.044
Yes	1 (6.3)*	5 (35.7)*		
No	15 (93.4)*	9 (64.3)*		

*GVHD* graft-versus-host disease, *IPFD* Invasive pulmonary fungal disease, *EBV* Epstein-Barr virus, *CMV* Cytomegalovirus; *Non-relapse mortality and relapse mortality as a percentage of total deaths

### Prognosis in the MSD and AD groups

Nineteen and 17 patients experienced disease relapse in the MSD and AD groups, respectively. The recurrence rates were significantly higher in the MSD than in the AD group (*P *< 0.05). Sixteen and 14 patients died in the MSD and AD groups, respectively. Thus, the mortality rate was significantly higher for the MSD than the AD group (*P *< 0.05). In addition, the proportion of recurrence among total deaths was significantly higher for the MSD than for the AD group (*P *< 0.05) (Table 2).

### Survival analysis in the MSD and AD groups

**Fig. 1:**
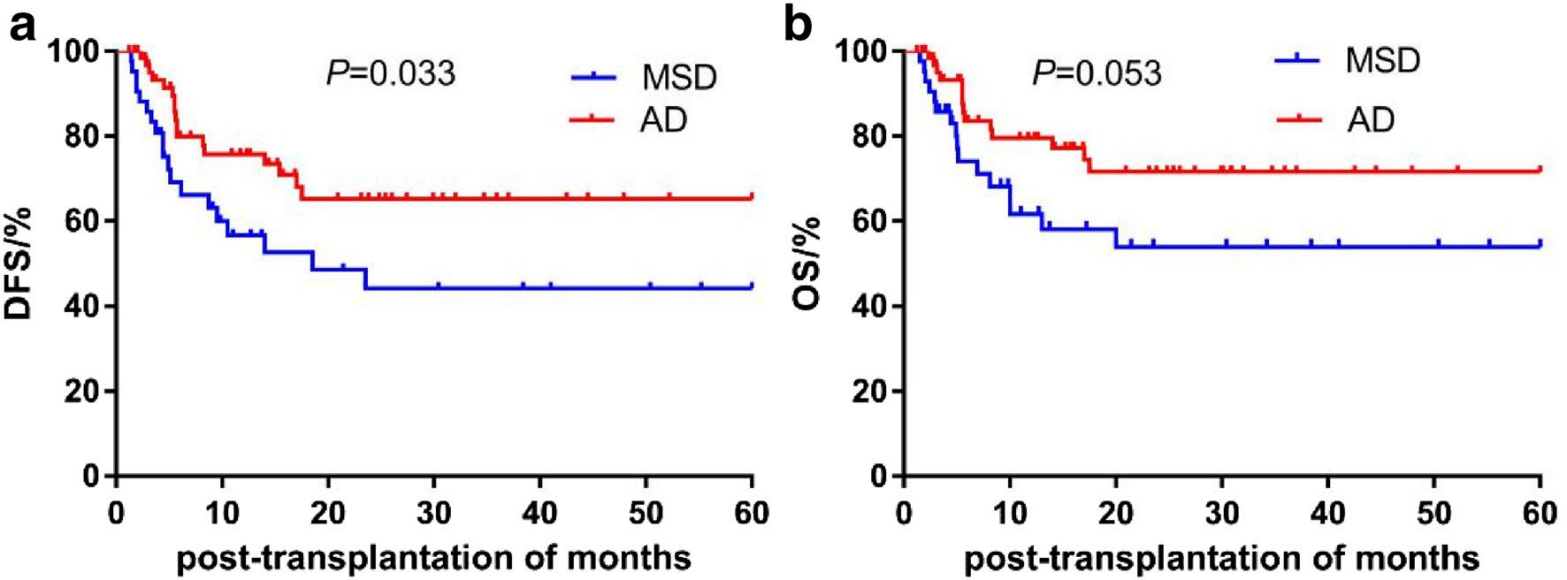
**a** Comparison of 5-year DFS rate between MSD and AD; **b** 5-year OS rate between MSD and AD

**Fig. 2:**
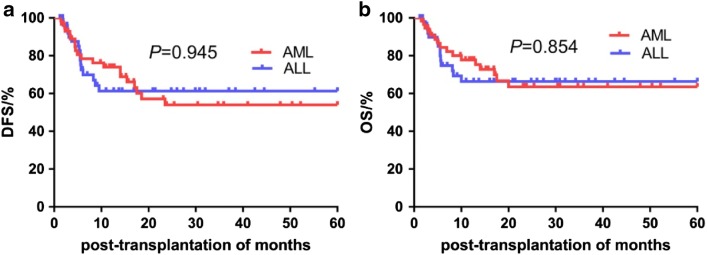
**a** Comparison of 5-year DFS rate between AML and ALL; **b** 5-year OS rate between AML and ALL

We compared the 5-year cumulative survival rates among patients with MSD and AD. The 5-year DFS was significantly higher in the AD than in the MSD group (65.2% vs. 43.3%, *P *= 0.033). The 5-year OS was also higher in the AD than in the MSD group, but without statistical significance (71.6% vs. 53.8%, *P *= 0.053) (Table 3, Fig. 1). Moreover, there were no significant differences in the 5-year DFS and OS among patients with AML and ALL (all *P *> 0.05) (Fig. 2). There were no significant differences in complications between AML and ALL except for EBV infection (*P *= 0.026) (Table 4).

**Table 3:** 5-year cumulative survival rate of all patients between MSD and AD

Variables	5-year cumulative OS		5-year cumulative DFS	
	Rate (%)	Mean time of survival (month)	Rate (%)	Mean time of survival (month)
MSD	53.8 ± 0.09	35.58 ± 4.62	43.3 ± 9.2	30.89 ± 4.63
AD	71.6 ± 0.07	45.32 ± 3.37	65.2 ± 7.1	42.09 ± 3.59
*P*-value	0.053		0.033

**Table 4:** Complications between AML and ALL

Group	aGVHD	Grade II-IVaGVHD	cGVHD	IPFD	Hemorrhagic cystitis	EBV infection	CMV infection
AML (n, %)	23 (39)	6 (10.2)	12 (20.3)	17 (28.8)	15 (25.4)	9 (15.3)	22 (37.3)
ALL (n, %)	21 (40.4)	8 (15.4)	13 (25)	18 (34.6)	18 (34.6)	18 (34.6)	26 (50)
*P*	1	0.568	0.651	0.545	0.307	0.026	0.186

*GVHD* graft-versus-host disease, *IPFD* invasive pulmonary fungal disease, *EBV* Epstein–Barr virus, *CMV* cytomegalovirus

**Fig. 3:**
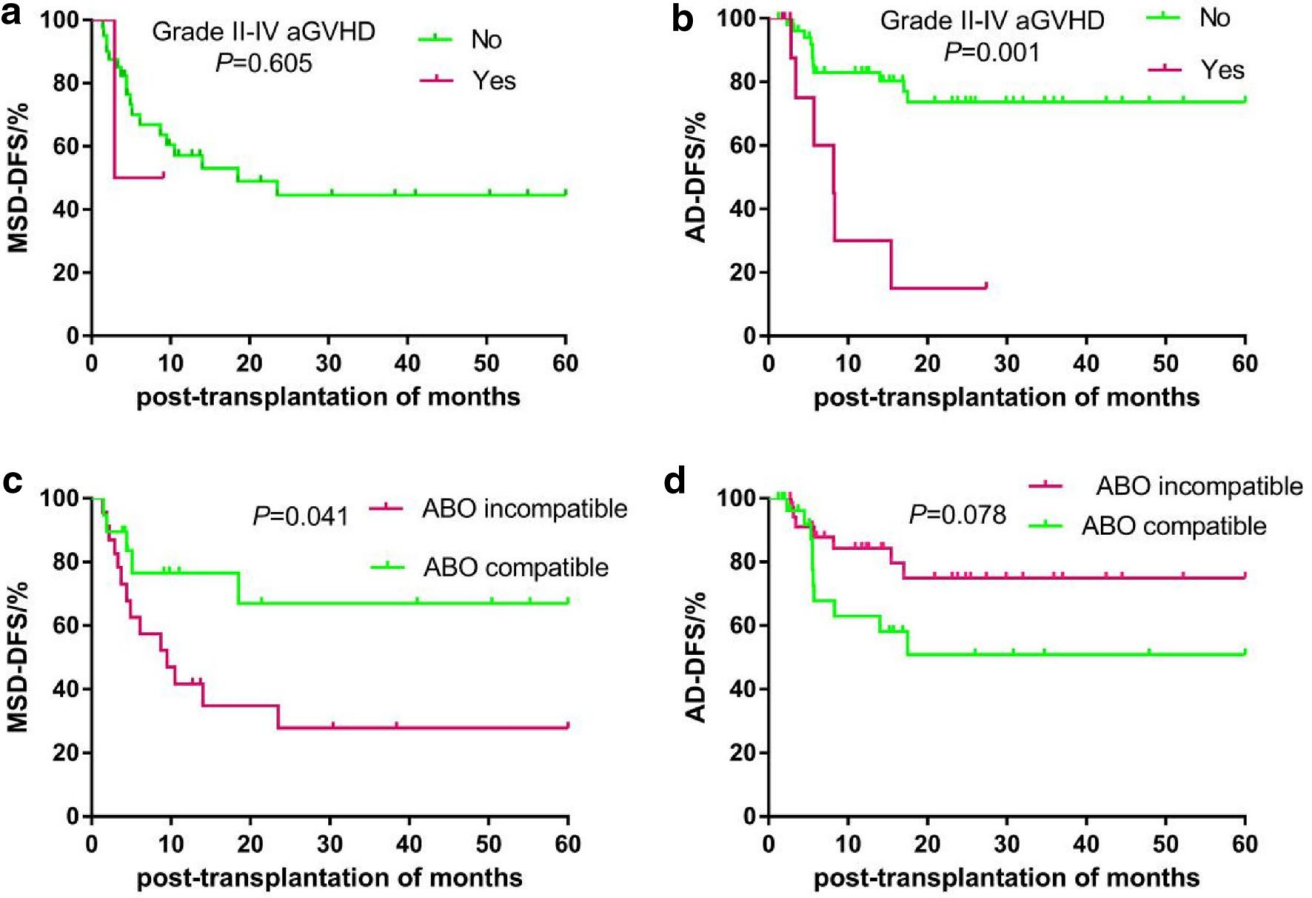
The impacts of grade II–IV aGVHD (**a**, **b**) and donor-recipient ABO compatibility (**c**, **d**) on 5-year DFS between MSD and AD in one hundred eleven children with acute leukemia

**Fig. 4:**
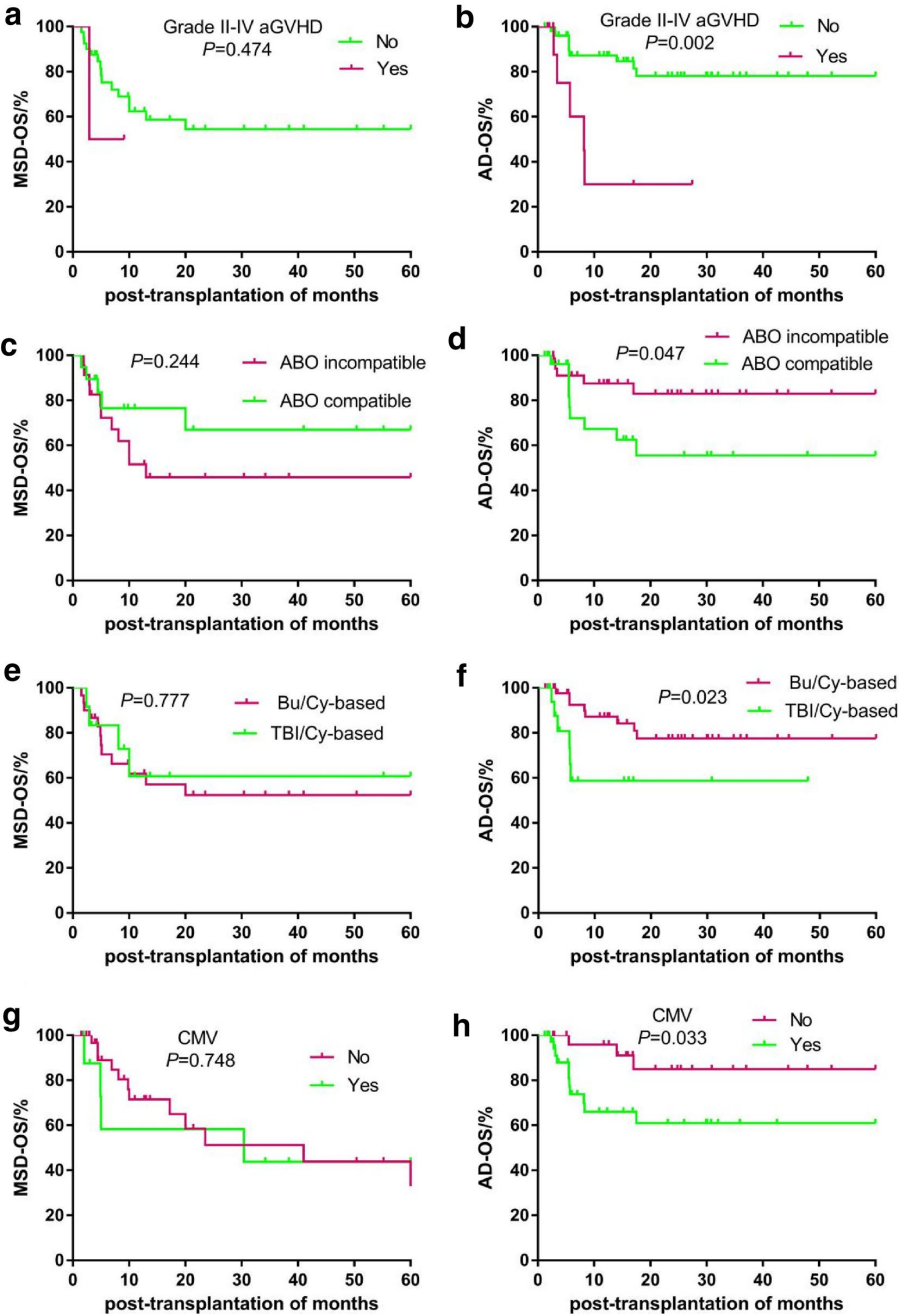
The factors of affecting 5-year OS rate between MSD and AD in one hundred eleven childhood acute leukemia; (**a**, **b** grade II–IV aGVHD; **c**, **d** donor-recipient ABO compatibility; **e**, **f** conditioning regimen; **g**, **h** CMV infection)

In addition, we separately assessed the factors affecting the survival of patients in the MSD and AD groups. The 5-year OS (78.1% vs. 30.0%, *P *= 0.002) and DFS (73.6% vs. 15.0%, *P *= 0.001) rates were significantly higher among patients without grade II–IV aGVHD in the AD group than among patients with grade II–IV aGVHD. The 5-year OS of patients who were donor-recipient ABO-compatible was significantly lower than that of those who were ABO-incompatible in the AD group (55.6% vs. 83.0%, *P *= 0.047). Moreover, the 5-year OS was significantly lower inpatients who received the TBI/Cy-based regimen than in those who received the Bu/Cy-based regimen in the AD group (58.7% vs. 77.6%, *P *= 0.023). The 5-year OS was significantly lower inpatients with CMV infection than in those without in the AD group (60.9% vs. 85.0%, *P *= 0.033). All these factors had no significant effect on the 5-year OS among patients in the MSD group (all *P *> 0.05). The 5-year DFS rate was significantly higher among the patients who were donor-recipient ABO-compatible than among those who were ABO-incompatible in the MSD group (67.0% vs. 27.7%, *P *= 0.041) (Table 5, Figs. 3 and 4). The other factors had no effect on the survival of patients between the MSD and AD groups.

**Table 5:** Factors of affecting 5-year cumulative survival rate between MSD and AD

Variables	5-year cumulative OS		5-year cumulative DFS	
	MSD	AD	MSD	AD
Grade II-IV aGVHD				
Yes	50.0 ± 35.4	30.0 ± 17.5	50.0 ± 35.4	15.0 ± 13.8
No	54.4. ± 9.0	78.1 ± 6.7	44.4 ± 9.3	73.6 ± 7.1
*P*-value	0.474	0.002	0.605	0.001
Donor-recipient ABO compatibility				
Compatible	66.6 ± 12.8	55.6 ± 11.4	67.0 ± 12.8	50.9 ± 11.6
Incompatible	45.9 ± 11.3	83.0. ± 7.1	27.7 ± 10.9	74.9. ± 8.5
*P*-value	0.244	0.047	0.041	0.078
Conditioning regimen				
Bu/Cy-based	52.3 ± 10.3	77.6 ± 7.1	44.1 ± 10.4*	69.2 ± 7.9*
TBI/Cy-based	60.8 ± 15.8	58.7. ± 13.1	40.5 ± 19.6*	58.7 ± 13.1*
*P*-value	0.777	0.023	0.854	0.093
CMV infection				
Yes	44.4 ± 18.9	60.9 ± 9.5	15.6 ± 14.2*	57.4 ± 9.7*
No	57.2 ± 9.7	85.0. ± 8.2	54 ± 9.8*	75 ± 9.9*
*P*-value	0.748	0.033	0.235	0.083

* Estimated value censored

### Univariate and multivariate Cox regression analyses of all childhood acute leukemia after allo-HSCT

Univariate Cox regression analysis showed that grade II–IV aGVHD (HR: 3.016, 95% confidence interval [CI] 1.217–7.473, *P *= 0.017) influenced the 5-year OS of childhood acute leukemia after allo-HSCT. The donor source (MSD) (HR: 2.035, 95% CI 1.057–3.920, *P *= 0.034) and grade II–IV aGVHD (HR: 2.914, 95% CI 1.261–6.736, *P *= 0.012) influenced the 5-year DFS.

The multivariate Cox regression analysis showed that grade II–IV aGVHD (HR: 3.524, 95% CI 1.310–10.988, *P *= 0.030) was an independent predictor of the 5-year OS for childhood acute leukemia after allo-HSCT. The donor source (MSD) (HR: 2.836, 95% CI 1.179–6.823, *P *= 0.020) and grade II–IV aGVHD (HR: 3.731, 95% CI 1.332–10.454, *P *= 0.012) were independent predictors of the 5-year DFS.

## Discussion

High-risk R/R childhood acute leukemia has a poor prognosis; allo-HSCT may provide effective treatment for afflicted patients [19–21]. In recent years, AD transplantation has made great progress in the treatment of childhood hematologic diseases [22–26]. Particularly, the 5-year OS and DFS of patients with childhood high-risk acute leukemia after unrelated bone marrow transplantation can reach up to 75% and 69.6%, respectively [27]. Our research was mainly focused on AD-PBSCT in the treatment of high-risk R/R childhood acute leukemia. In this study, we found that the 5-year OS and DFS rates after AD-PBSCT were 71.6% and 65.2%, respectively. Its associated long-term survival was similar to that of bone marrow transplantation. In addition, we found that the 5-year DFS was significantly higher in the AD than in the MSD group (65.2% vs. 43.3%, *P *= 0.033). The 5-year OS rate was also higher in the AD than in the MSD group, but without statistical significance (71.6% vs. 5 3.8%, *P *= 0.053). We also found that the recurrence rate and the proportion of recurrence among total deaths were significantly higher in the MSD than in the AD group (*P *< 0.05). Zheng et al. also suggested that for high-risk or advanced childhood acute leukemia, unrelated transplantation yielded a similar long-term survival, but a better anti-leukemic effect than MSD [23]. In terms of survival, AD-PBSCT showed a certain advantage over MSD transplantation. In addition, Keating et al. believed that umbilical cord blood may be also a great alternative cell source when there was no MSD, but further prospective research is needed [28].

Transplantation related complications are the main determinants of survival; particularly, GVHD may be one of the most important factors affecting survival and prognosis [29–31]. We compared the occurrence of complications between the AD and MSD groups. The cumulative incidence of grade II–IV aGVHD was higher in theAD than in the MSD group, but without statistical significance (*P *= 0.052). Currently, the application of donor regulatory T cells ameliorates the clinical and histologic symptoms of aGVHD and significantly enhances survival. It prevents aGVHD and it is effective for the treatment of life-threatening complications [31]. The cumulative incidences of EBV and CMV infections were significantly higher in the AD than in the MSD group (all *P *< 0.001). Our study also showed that the 5-year OS was significantly lower in patients with CMV infection than in those without (60.9% vs. 85.0%, *P *= 0.033) in the AD group. However, in the MSD group, EBV infection had no significant effect on the survival in the AD and MSD groups. Previous studies have shown that CMV reactivation remains a risk factor for poor post-transplantation outcomes and there are no preventive measures specifically for the recurrence of hematological diseases [32], but the impact of CMV infection on survival after transplantation remains controversial [33]. In addition, rituximab and donor lymphocyte infusion are established as successful options for EBV infection [34]. Therefore, EBV and CMV infections do not always threaten the lives of patients. In this study, there were no significant differences in the incidences of IPFD, hemorrhagic cystitis, cGVHD, and other complications.

We assessed the factors affecting the survival of patients in the MSD and AD groups. We found that the 5-year OS (78.1% vs. 30.0%, *P *= 0.002) and DFS (73.6% vs. 15.0%, *P *= 0.001) rates were significantly higher among patients without grade II–IV aGVHD in the AD group than among patients with grade II–IV aGVHD. This is consistent with previous studies that showed that grade II–IV aGVHD is a key factor that affects survival and prognosis among patients [35–38]. The 5-year OS rate was significantly lower among donor-recipient ABO-compatible than among ABO-incompatible patients in the AD group(55.6% vs. 83.0%, *P *= 0.047). However, the 5-year DFS rate was significantly higher among donor-recipient ABO-compatible than among ABO-incompatible patients in the MSD group (67.0% vs. 27.7%, *P *= 0.041). At present, the impact of donor and recipient ABO statuses on the efficacy of transplantation among leukemia patients remain controversial. Wang et al. showed that donor-recipient ABO in compatibility was significantly correlated with delayed platelet recovery among older donors and higher transplantation related mortality rates and higher rates of grade III aGVHD [39]. Nicolas et al. revealed that the major ABO incompatibility was associated with a significantly low recurrence rate(HR = 0.65, *P *= 0.04) [40]. However, some other studies showed that donor-recipient ABO mismatch had no significant effects on major survival outcomes after allo-HSCT, such as the incidence of GVHD, rates of relapse and mortality, DFS, and OS. Also, donor-recipient ABO incompatibility was not associated with delayed platelet and neutrophil engraftment after allo-HSCT [41, 42]. There was no evidence of a substantial effect of donor-recipient ABO incompatibility on the outcome of allo-HSCT among patients with leukemia [43]. The Japanese Marrow Donor Program reported that the 1-year survival rate after ABO-matched transplantation was 63%, compared with 57% after minor and major ABO mismatched transplantation. Therefore, donor-recipient ABO matching has a modest effect on survival [44]. The 5-year OS rate was significantly lower among patients who received the TBI/Cy-based regimen (58.7% vs. 77.6%, *P *= 0.023) than among those who received the Bu/Cy-based regimen in the AD group. This may be related to the application of TBI to increase the risk of grade II–IV aGVHD, which in turn affects the survival of patients [45, 46]. Grade II–IV aGVHD, conditioning regimen, and donor-recipient ABO status had no significant effect on 5-year OS among patients in the MSD group (all *P *> 0.05).

In addition, we comprehensively assessed all children with leukemia. The univariate Cox regression analysis showed that grade II–IV aGVHD (HR: 3.016, 95% CI 1.217–7.473, *P *= 0.017) was a factor that influenced the 5-year OS of childhood acute leukemia after allo-HSCT. This result is consistent with the factor affecting AD transplantation. The donor source (MSD) (HR: 2.035, 95% CI 1.057–3.920, *P *= 0.034) and grade II–IV aGVHD (HR: 2.914, 95% CI 1.261–6.736, *P *= 0.012) were factors that influenced the 5-year DFS. The multivariate Cox regression analysis showed that grade II–IV aGVHD (HR: 3.524, 95% CI 1.310–10.988, *P *= 0.030) was an independent risk factor of 5-year OS. The donor source (MSD) (HR: 2.836, 95% CI 1.179–6.823, *P *= 0.020) and grade II–IV aGVHD (HR: 3.731, 95% CI 1.332–10.454, *P *= 0.012) were independent predictors of 5-year DFS. Chen et al. also showed that grade III–IV aGVHD may be related to worse survival, but cGVHD had no significant influence on DFS or OS [37]. Tomizawa et al. also showed that grade II–IV aGVHD (*P *= 0.049) was related to inferior OS [47]. In our study, the disease status at HSCT had no significant effect on survival. Montoro et al. found that there were no obvious differences in OS, DFS, and recurrence rates between patients transplanted in CR1 and CR2. However, patients with high-risk cytogenetics at diagnosis tended to have significantly worse prognoses [48]. In addition, a previous study showed that the 3-year OS rates among patients who underwent transplants at CR1 and CR2 were 73% and 25%, respectively. This study supported the notion that allo-HSCT maybe a suitable treatment for high-risk AML at CR1 [19].

A previous study has reported that the 3-year OS rates among patients who underwent allo-HSCT and those who only underwent salvage chemotherapy were 67% and 12%, respectively. In addition, the 5-year OS rates among patients who underwent allo-HSCT and those who only underwent salvage chemotherapy were 44% and 4%, respectively (*P *< 0.001). Allo-HSCT remains the most promising treatment option among patients with refractory AML [49]. Xue et al. found that haploidentical HSCT (haplo-HSCT) only showed a significant survival advantage among high-risk ALL patients. The authors posited that haplo-HSCT can be used as an alternative treatment modality for high-risk ALL patients [20]. A study conducted in China showed that allo-HSCT can be recommended as treatment for intermediate-risk and high-risk AML-CR1, some low-risk AML-CR1, Ph^+^ ALL, high-risk ALL, and adult standard-risk ALL-CR1. The effects of AD-HSCT and MSD-HSCT are comparable in China [50]. Haplo-HSCT can achieve similar results as MSD-HSCT in high-risk ALL patients with CR1, and this transplant maybe an effective option for post-remission treatment of high-risk ALL-CR1 patients without MSD [21, 50].

## Conclusions

AD-PBSCT is effective for high-risk R/R childhood leukemia. The incidence of complications is low, and these complications have no significant effect on survival. These results all show that AD-PBSCT may have better clinical outcomes than MSD-PBSCT. Overall, AD-PBSCT can be used as first-line treatment for high-risk R/R childhood leukemia; its efficacy and safety may even be better than those of MSD transplantation.

## Data Availability

All data generated or analyzed during this study are included in this published article.

## Abbreviations

AD: Alternative donor
ALL: Acute lymphoblastic leukemia
AML: Acute myeloid leukemia
DFS: Disease-free survival
DLI: Donor lymphocyte infusion
GVHD: Graft-versus-host disease
aGVHD: Acute graft-versus-host disease
cGVHD: Chronic graft-versus-host disease
IPFD: Invasive pulmonary fungal disease
MSD: Matching-sibling donor
OS: Overall survival
TBI: Total body irradiation
CMV: Cytomegalovirus
CR: complete remission
CsA: Cyclosporine A
EBV: Epstein–Barr virus
HSCT: Hematopoietic stem cell transplantation
haplo-HSCT: Haploidentical hematopoietic stem cell transplantation
